# Intraspecific differences in tolerance to elevated pH and alkalinity in brook stickleback (*Culaea inconstans*) inhabiting neutral and alkaline lakes

**DOI:** 10.1242/jeb.251351

**Published:** 2026-07-09

**Authors:** Alex M. Zimmer, Charles Bernard, Marina Giacomin, Anne-Marie Dion-Côté, Greg G. Goss, Chris N. Glover

**Affiliations:** ^1^ Department of Biological Sciences, University of New Brunswick, Saint John, NB, Canada, E2L 4L5; ^2^ Department of Biological Sciences, University of Alberta, Edmonton, AB, Canada, T6G 2E9; ^3^ Département de Biologie, Université de Moncton, Moncton, NB, Canada, E1A 3E9; ^4^ Core Geoscience Services Inc., Whitehorse, YT, Canada, Y1A 1C5; ^5^ Faculty of Science and Technology and Athabasca River Basin Research Institute, Athabasca University, Athabasca, AB, Canada, T9S 3A3

**Keywords:** Ammonia excretion, Ion regulation, Nitrogen, Stress

## Abstract

This study aimed to characterize mechanisms of tolerance to elevated pH and carbonate alkalinity in brook stickleback (*Culaea inconstans*), a fish species with a broad pH habitat range, including highly alkaline waters such as Buffalo Lake (pH 9.2; [HCO_3_^−^] 20.0 mmol l^−1^) in Alberta, Canada. Stickleback from Buffalo Lake and a more pH-neutral, low-alkalinity reference lake (Buck Lake; pH 8.2; [HCO_3_^−^] 3.0 mmol l^−1^) were collected from the wild and acclimated to common conditions (pH 8.0; [HCO_3_^−^] 1.7 mmol l^−1^) for 2 months. Both populations were then exposed to alkaline conditions (pH 9.5; [HCO_3_^−^] 22.1 mmol l^−1^) in the laboratory, resulting in a significant decrease in survival (14% by 7 days of exposure) in Buck Lake fish, but no mortality in Buffalo Lake stickleback. In a 4 day alkaline exposure, no significant differences in nitrogen or ion regulation were observed between populations, but a trend towards reduced whole-body chloride concentration in Buck Lake stickleback suggested potential disruptions in ion and acid–base regulation in this population. In Buck Lake stickleback, genes involved in cellular stress and immune responses were differentially regulated in gill tissue in response to alkaline exposure, while this response was largely absent in Buffalo Lake stickleback. Overall, alkaline tolerance is higher in brook stickleback resident in an alkaline lake compared with those from a neutral lake, but tolerance does not appear to be a function of differences in nitrogen or ion/acid–base regulation. Rather, alkaline-tolerant stickleback mounted a relatively weaker transcriptomic stress response in the gill, which might represent transcriptional dampening that confers increased tolerance.

## Teaser

**Summary:** Brook stickleback from a naturally alkaline, high pH lake are uniquely tolerant of elevated pH. Stickleback from a neutral reference lake were intolerant of the same high pH conditions, exhibiting mortality and a cellular stress response that was absent in the alkaline lake stickleback population.

## INTRODUCTION

Alkaline saline lakes are a relatively common feature of arid regions and are characterized by a permanently high alkalinity, generally resulting from high concentrations of HCO_3_^−^ (ca. 13–67 mmol l^−1^), and pH values ranging from 9.0 to 10.0 (Brauner et al., 2013; Danulat, 1995; Wilkie and Wood, 1996; Wood, 2022). Exposure to elevated pH (pH >9.0) is generally very challenging in fish and results in disturbances in nitrogen, acid–base and ion balance (Brauner et al., 2013; Danulat, 1995; Wilkie and Wood, 1996; Wood, 2022). In most fishes, 80–90% of nitrogenous waste arising from protein catabolism is excreted across the gills as ammonia, with urea accounting for the majority of the remaining proportion (Wood, 1993). The current consensus is that ammonia excretion occurs as facilitated NH_3_ diffusion and that this process relies on an outwardly directed NH_3_ partial pressure (*P*_NH_3__) gradient that is supported by an acid-trapping mechanism in the apical gill boundary layer whereby NH_3_ is titrated to NH_4_^+^ (Wright and Wood, 2009; Wright et al., 1986; Zimmer, 2024). In alkaline water, when pH is near or exceeds the p*K*_a_ of the NH_3_⇌NH_4_^+^ equilibrium reaction (p*K*_a_≈9.0–9.5), the *P*_NH_3__ gradient is greatly diminished, or even reversed, resulting in a reduction in ammonia excretion and an increase in the concentration of ammonia in the blood plasma and tissues (Kwan et al., 2024; McGeer and Eddy, 1998; Thompson et al., 2016; Wilkie and Wood, 1991; Wilkie et al., 1993, 1996; Wright et al., 1993; Zimmer et al., 2024).

While the most obvious effects of alkaline water on fish are related to the impact of pH on nitrogen regulation, it can also have effects on acid–base balance. Alkaline water has been described as a ‘CO_2_ vacuum’ (Johansen et al., 1975) because exposure to alkaline water results in a large, outwardly directed *P*_CO_2__ gradient such that fish exposed to alkaline conditions experience a respiratory alkalosis (McGeer and Eddy, 1998; Wilkie and Wood, 1991; Wilkie et al., 1996; Wright and Wood, 1985). In some instances, pH can be maintained in the face of reduced plasma *P*_CO_2__ via an increase in metabolic proton production (Wilkie et al., 1996), although this likely depends on water chemistry parameters such as [Ca^2+^] and [HCO_3_^−^] (Wilkie and Wood, 1996; Yesaki and Iwama, 1992). Fish exposed to high pH also experience a disruption in ion balance (Kwan et al., 2024; McGeer and Eddy, 1998; Thompson et al., 2015; Wilkie and Wood, 1991; Wilkie et al., 1993; Yesaki and Iwama, 1992; Zimmer et al., 2024) due to the inhibitory effects of high pH on ion influx rates (Scott et al., 2005; Wilkie et al., 1999; Wright and Wood, 1985). However, the effect of high pH on ion balance appears to be variable (Scott and Wilson, 2007). The mechanism underlying the inhibitory effect of high pH on ion uptake is currently not well understood but may involve a reduction in the availability of H^+^ and HCO_3_^−^ as counter ions for Na^+^ and Cl^−^ uptake, respectively, in response to respiratory alkalosis (Wilkie et al., 1999; Wood, 2022).

Despite the physiological challenges of life in alkaline waters, some examples exist of fishes that inhabit waters of extremely high pH. These include the Magadi tilapia, *Alcolapia grahami* (Randall et al., 1989), in Lake Magadi, Kenya (pH 9.6–10.0) and the tarek (pearl mullet), *Alburnus tarichi* (Danulat and Kempe, 1992), in Lake Van, Turkey (pH 9.8). In more moderately alkaline lakes (pH <9.5), a greater diversity of species has been observed (McGeer et al., 1994; Mitchell and Prepas, 1990). This capacity of some fish species and populations to inhabit lakes of extreme alkalinity and pH results in a ‘natural experiment’ for comparative physiologists interested in understanding the mechanisms that underlie tolerance to alkaline waters. For example, life in extreme alkaline conditions has resulted in unique adaptations to counteract the physiological disturbances associated with exposure to high pH/alkalinity. Perhaps the most notable case is that of the Magadi tilapia, which circumvents the inhibitory effect of its highly alkaline environment on ammonia excretion rates by excreting 100% of its nitrogenous waste as urea (Randall et al., 1989; Wood et al., 1989). Magadi tilapia express the full suite of ornithine urea cycle (OUC) enzymes as adults (Lindley et al., 1999; Randall et al., 1989), leading to a fully ureotelic physiology that appears to be obligatory (Wood, 2022).

Other species inhabiting less alkaline environments, such as Pyramid Lake in Nevada, USA (pH ∼9.4), show less dramatic adaptations, including a reduction in nitrogen production and a shift to renal ammonia excretion (McGeer et al., 1994; Wilkie et al., 1993, 1994; Wright et al., 1993), as well as a greater capacity to regulate ion and acid–base balance (Wilkie et al., 1994). Tolerance of elevated plasma and tissue ammonia levels may also be critical to survival in alkaline waters in some species such as the naked/scaleless carp (*Gymnocpyris przewalskii*), which inhabits the alkaline Qinghai Lake (pH ∼9.3) in the Tibetan plateau (Wood, 2022). This species demonstrates much higher activity of glutamine synthetase and glutamate dehydrogenase, which catalyse ammonia-consuming reactions, compared with alkaline-naive species (Wang et al., 2003; Wood, 2022). Moreover, several studies have identified a large number of genes related to nitrogen metabolism, ion regulation, acid–base regulation and immune responses that appear to be under selection in species inhabiting alkaline lakes (Tong and Li, 2020; Tong et al., 2021; Xu et al., 2017; Zhang et al., 2015; Zhou et al., 2023).

While there are many cases of evolved alkaline tolerance in select fish species, some of which are endemic to a particular alkaline lake, few studies have assessed whether intraspecific differences in alkaline tolerance exist between neutral and alkaline populations of a single species. These differences in tolerance might allow a given species to inhabit alkaline lakes, effectively expanding its ‘chemical niche’ (Zimmer et al., 2021). Therefore, the goals of the present study were to assess the alkaline tolerance of fish inhabiting a naturally alkaline endorheic lake, Buffalo Lake (pH 9.2), in Alberta, Canada, and to identify the potential mechanisms associated with this phenomenon. We collected brook stickleback (*Culaea inconstans*) from Buffalo Lake and a more pH-neutral reference lake (Buck Lake; pH 8.2), and acclimated fish from both populations to common neutral laboratory conditions (pH 8.0) for over 2 months. We hypothesized that Buffalo Lake stickleback would have increased alkaline tolerance compared with Buck Lake stickleback and that this tolerance would be a result of intraspecific differences in the physiological response to alkaline exposure. Based on this hypothesis, we predicted that Buffalo Lake stickleback, relative to Buck Lake stickleback, would exhibit smaller disruptions in nitrogen and ion balance in response to laboratory exposure to alkaline water (pH 9.5).

Furthermore, we predicted that any potential changes in the branchial activity of enzymes that play roles in correcting alkaline-induced disruptions in nitrogen, ion and acid–base regulation (Na^+^/K^+^-ATPase, H^+^-ATPase and carbonic anhydrase) would be greater in Buffalo Lake fish, reflecting a higher capacity to regulate physiological homeostasis under alkaline conditions. This prediction was based on previous evidence demonstrating the importance of branchial regulation of ion and nitrogen balance in response to alkaline exposure in other species (Laurent et al., 2000; Wilkie et al., 1994; Zhao et al., 2024). We further predicted that these population-specific differences in the regulation of enzyme activity would also be reflected by differences in transcriptomic responses in the gill. Transcriptomic analyses also allowed for the identification of other potential cellular responses in the gills of brook stickleback that might be important for alkaline tolerance.

## MATERIALS AND METHODS

### Fish

Brook stickleback, *Culaea inconstans* (J. P. Kirtland 1840), were collected from a neutral lake (Buck Lake; 52.992878, −114.714775; mean±s.e.m. mass 1.29±0.05 g, range 0.82–1.64 g) and an alkaline lake (Buffalo Lake; 52.530261, −112.815954; mean±s.e.m. mass 1.01±0.04 g, range 0.81–1.54 g) in Alberta, Canada [Alberta Fish Research Licence #21–3801] in September and October 2021 using standard minnow traps baited with commercial pet food. The chemical compositions of Buffalo Lake and Buck Lake water at the time of fish collection, in addition to historical values, are presented in Table 1. Fish were transferred from the traps to coolers containing aerated lake water held within 2°C of the water temperature at the site (temperature range at sites 10–15°C). Coolers were transported to the University of Alberta aquatics facilities and fish were subsequently transferred to 100 l acrylic tanks filled with approximately 20 l of lake water. The tanks were fitted with air stones and supplied with facility water (Table 1; 10–12°C) at a rate of approximately 1 l min^−1^ such that the lake water in the tanks was gradually replaced with facility water. Stickleback from both populations were held in flow-through facility water (10–12°C) under a 12 h:12 h light:dark photoperiod and fed twice daily (10:00 h and 15:00 h) to satiation with live brine shrimp. Mortality under these conditions was less than 5% overall for both populations. Fish were acclimated for at least 2 months prior to experimentation to reduce the potential effects of acclimation to alkaline water in Buffalo Lake fish on the subsequent response to alkaline exposure in the lab. Fish were fasted for 24 h prior to all experimental procedures described below and remained fasted during experiments. Handling and experimentation were approved by the University of Alberta Biosciences Animal Care Committee (Animal Utilization Protocol 00003831).

**Table 1.** Chemical composition of lake water and lab exposure water

Sampling	pH	[HCO_3_^−^] (mmol l^−1^)	[Na^+^] (mmol l^−1^)	[Cl^−^] (mmol l^−1^)	[Ca^2+^] (mmol l^−1^)
At capture					
Buck Lake	7.3	1.6	0.6	0.08	0.9
Buffalo Lake	9.3	21.0	26.1	1.1	0.3
Historical					
Buck Lake	8.2 (6.6–9.4) *n*=514	3.0 (0.1–3.0) *n*=45	0.6 (0.4–1.0) *n*=45	0.1 (0.03–0.1) *n*=44	0.6 (0.5–0.8) *n*=44
Buffalo Lake	9.2 (8.8–9.5) *n*=92	20.0 (13.8–20.6) *n*=92	23.6 (16.8–30.0) *n*=92	0.5 (0.3–1.0) *n*=92	0.2 (0.1–0.4) *n*=92
Facility water	8.0	1.7±0.1 *n*=8	0.6±0.1 *n*=8	0.2±0.01 *n*=8	1.2±0.1 *n*=8
Alkaline water	9.5	22.1±0.3 *n*=8	24.48±0.66 *n*=8	0.2±0.02 *n*=8	0.1±0.01 *n*=8

Buffalo and Buck Lake data are values measured in water samples collected at the time of fish capture and means of samples collected over 40 years of historical sampling (data obtained from the Government of Alberta Water Quality Portal, accessed 16 July 2024: https://environment.extranet.gov.ab.ca/apps/WaterQuality/dataportal/), with range included in parentheses. Facility and alkaline water data are presented as means±s.e.m of measurements made in the lab.

### Survival in alkaline water

Tolerance of high pH and high carbonate alkalinity water (hereafter referred to as ‘alkaline water/conditions’) was tested in Buck Lake and Buffalo Lake stickleback. Alkaline water approximating the water chemistry of Buffalo Lake (Table 1) was achieved by adding 20 mmol l^−1^ NaHCO_3_ to facility water and adjusting the pH to 9.5 using 1 mol l^−1^ NaOH. Note that alkaline water was prepared at least 12 h prior to fish exposure to allow carbonate precipitates to settle out of solution. Fish were transferred in groups of 6–8 to 5 l static aquaria containing 4 l of alkaline water that were held in a 10–12°C water bath. Each aquarium housing 6–8 individuals was treated as a single replicate sample, with 3–4 replicate aquaria per population (Buck Lake: *n*=4 replicate aquaria; Buffalo Lake: *n*=3 replicate aquaria). Aquaria were fitted with automatic titrators (pH Controller, BlueLab, Tauranga, New Zealand) that maintained pH at 9.5±0.1 via the addition of 0.1 mol l^−1^ NaOH. Water changes (80% of total volume) were conducted each day, and fish were fasted for the duration of the 7 day exposure period. Throughout the exposure, fish were checked for signs of morbidity (primarily the loss of equilibrium), at which point they were euthanized using 1 g l^−1^ of neutralized MS-222. Fish that died prior to being euthanized (i.e. mortalities) were removed from the tank. Daily morbidities and mortalities were recorded and used to determine survival rates in alkaline water. Note that for each population, one control survival experiment (6–8 individuals in 1 replicate tank) using normal facility water (pH 8.0) was conducted in order to ensure that mortality was not a function of handling unrelated to the alkaline exposure.

### Acute alkaline exposures

Two sets of additional experiments were conducted to determine population-specific effects of acute exposure to alkaline water for 1 or 4 days. For the 4 day experiment, nitrogen excretion rates were measured over the duration of the exposure, whereas nitrogen excretion rates were not measured in the 1 day exposure, which also included a 1 day control exposure. In the 4 day experiment (Buck Lake: *n*=7; Buffalo Lake: *n*=7), fish were transferred individually to 200 ml black opaque plastic containers held in a 10–12°C water bath. Each container was fitted with tubing that supplied the containers with recirculating facility water held at 10–12°C and an air line to maintain constant aeration. Fish were held under these recirculating conditions overnight. The following morning, a 24 h measurement period (i.e. the period over which water samples were collected for later determination of ammonia and urea excretion rates) in control facility water was conducted. For this measurement period, recirculating flow was stopped and water in the containers was lowered to a set volume (approximately 50 ml) and water samples (2 ml) were collected at 0 and 12 h. After this 12 h period, water was replaced by gently flushing the containers with fresh facility water and samples were collected again at 0 and 12 h post-water change (i.e. 12–24 h of the control measurement period). The containers were then flushed thoroughly with alkaline water (Table 1) that was prepared as described above. Complete water exchange of the containers was monitored by pH electrodes that were placed in each container. The alkaline measurement period began only once the pH of the water was equal to 9.5. Throughout the entire 4 day measurement period, pH was maintained at 9.5±0.1 by automatic titration (pH Controller, BlueLab) with 0.01 mol l^−1^ NaOH; each container was fitted with its own independent titration unit. Water samples were collected every 12 h, with water changes occurring every 12 h following the procedure described above. Water samples were stored at −20°C until later measurement of ammonia and urea concentrations, which were used to calculate excretion rates over 24 h periods (see ‘Analytical techniques and calculations’, below).

At the end of the 4 day period, fish were removed from the containers and water volume was recorded. Fish were then euthanized using 1 g l^−1^ MS-222 titrated to pH 9.5 using NaOH, weighed, and the entire gill basket was excised, divided into two halves, flash frozen in liquid nitrogen and stored at −80°C. One half of the gill basket was used in enzyme activity assays while the other half was used for RNA sequencing analyses (see ‘Transcriptomic analyses’, below). The viscera (gut, liver, kidney and gonads) were removed and discarded, and the remaining carcass of the fish was flash frozen in liquid nitrogen and stored at −80°C until subsequent measurement of tissue ammonia, urea, Na, Cl and lactate concentrations (see ‘Analytical techniques and calculations’, below). To assess endpoints at 1 day of exposure, the same procedure described above was additionally conducted on fish exposed to alkaline water for 1 day (Buck Lake: *n*=6; Buffalo Lake: *n*=8), or to facility water for 1 day as a control group (Buck Lake: *n*=8; Buffalo Lake: *n*=8). Note again that nitrogen excretion measurements were not conducted for these 1 day exposures.

### Analytical techniques and calculations

Total CO_2_ in water samples was measured using a 965 CO_2_ analyser (Corning Inc., Corning, NY, USA) and [HCO_3_^−^] was calculated using the Henderson–Hasselbalch equation and αCO_2_ and p*K*_app_ values derived from Boutilier et al. (1984). [Na^+^] and [Ca^2+^] in water samples were determined by atomic absorption flame spectroscopy (iCE 3500 AAS Atomic Absorption Spectrometer, ThermoFisher Scientific, Waltham, MA, USA) and [Cl^−^] was determined by a colorimetric assay (Zall et al., 1956).

[Ammonia] and [urea] in water samples were determined by colorimetric assays (Rahmatullah and Boyde, 1980; Verdouw et al., 1978). Ammonia and urea excretion rates were calculated using the following equation:

(1) $$\begin{array}{rl} & \mathrm{Ammonia}/\mathrm{urea}\;\mathrm{excretion}\;\mathrm{rate}\\ & \;=\frac{({\left[\mathrm{ammonia}/\mathrm{urea}\right]}_{f}-{\left[\mathrm{ammonia}/\mathrm{urea}\right]}_{i})\times \;V}{M\times \;\mathrm{\Delta t}}, \end{array}$$

where [ammonia/urea]_f_ and [ammonia/urea]_i_ are the final and initial concentrations of ammonia-N or urea-N (μmol N l^−1^), *V* is the volume of water in the container holding the fish (l), *M* is the mass of the fish (g) and Δ*t* is the change in time over the measurement period (h).

Carcass analytes were determined following methods described previously (Zimmer et al., 2024). Briefly, frozen carcass samples were ground to a fine powder in a liquid nitrogen-cooled mortar and pestle. The powdered samples were deproteinized by the addition of 5 volumes of 8% perchloric acid and incubation on ice for 5 min. Samples were then centrifuged for 2 min at 10,000 ***g*** to obtain a supernatant which was neutralized by the addition of 3 mol l^−1^ KOH. The concentration of ammonia in the supernatant was determined using a commercial kit (Sigma-Aldrich, St Louis, MO, USA), while urea concentration was determined using the same colorimetric assay described above for water samples (Rahmatullah and Boyde, 1980). Similar to water samples, [Na^+^] and [Cl^−^] in neutralized tissue digests were measured by flame atomic absorption spectroscopy and a colorimetric assay (Zall et al., 1956), respectively, and lactate concentration was determined by the lactate dehydrogenase method (Bergmeyer, 1983).

The activity of Na^+^/K^+^-ATPase, H^+^-ATPase and carbonic anhydrase in gill tissue was assayed following established protocols (Henry, 1991; Lin and Randall, 1993; McCormick, 1993) described previously (Lim et al., 2015). Frozen gill tissue was homogenized on ice in a sodium deoxycholate buffer containing EGTA and centrifuged for 5 min at 10,000 ***g*** to remove cellular debris and cartilage. Na^+^/K^+^-ATPase and H^+^-ATPase activity in the collected supernatant (10 μl) was assayed indirectly by the rate of decrease in NADH concentration (measured at 340 nm using a plate spectrophotometer; SpectraMAX, Molecular Devices, Menlo Park, CA, USA) over a 15 min period in the presence or absence of ouabain (Na^+^/K^+^-ATPase inhibitor) or *N*-ethyl-maleimide (NEM) (H^+^-ATPase inhibitor) with sodium azide. Protein concentration in the homogenates was determined using a commercial assay kit (Bio-Rad Protein Assay, Bio-Rad, Hercules, CA, USA) and ATPase activity was expressed as μmol ADP mg^−1^ protein h^−1^.

The activity of carbonic anhydrase in gill homogenates (10 μl) was determined by the rate of pH change over a 30 s period following the addition of 1 ml CO_2_-saturated water to 10 ml reaction buffer (pH 7.4) held at 2–4°C. Activity was determined by the difference between catalysed (homogenate added) and non-catalysed (no homogenate added) reaction rates. Protein concentration in the homogenates was determined using the same commercial assay kit described above and carbonic anhydrase activity was expressed as μmol H^+^ mg^−1^ protein min^−1^.

### Transcriptomic analyses

RNA was extracted from gill tissue of a random subset of Buck Lake and Buffalo Lake stickleback exposed to alkaline water for 4 days (Buck Lake: *n*=6; Buffalo Lake: *n*=6) or to control conditions (Buck Lake: *n*=6; Buffalo Lake: *n*=6). Frozen gill tissue (∼20–40 mg) was homogenized in 1 ml ice-cold Qiazol (Qiagen, Hilden, Germany) using a stator and rotor homogenizer. Total RNA was precipitated from the homogenate following the manufacturer's protocol. RNA was reconstituted in nuclease-free water (20–50 μl) and RNA concentration and quality were assessed using a bio-analyser (2100 Bioanalyzer, Agilent Technologies, Inc., Santa Clara, CA, USA). RNA integrity number (RIN) was >9 in all samples.

mRNA stranded library preparation (New England Biolabs, Ltd, Ipswich, MA, USA), quality control and RNA sequencing (NovaSeq 6000 PE100, Illumina, Inc., San Diego, CA, USA) were performed by Genome Quebec (Montreal, QC, Canada). Reads were then filtered and trimmed using *Trimmomatic* (v.038; Bolger et al., 2014) with default parameters, except for the use of HEADCROP:12 to account for remaining adapters after demultiplexing. Read quality was assessed using *FastQC* (v.0.11.9; Brown et al., 2017), and results were compiled with *MultiQC* (v.1.13; Ewels et al., 2016), which did not reveal any significant issues apart from high sequence duplication levels.

Trimmed reads were aligned to the three-spined stickleback (*Gasterosteus aculeatus*) reference genome (GCF_016920845.1) using *STAR* (v.2.7.7; Dobin et al., 2013). The *outFilterScoreMinOverLRead* and *outFilterMatchNminOverLread* parameters were relaxed to 0.4 and 0.3, respectively, to account for divergence between *C. inconstans* and *G. aculeatus*. Sorted reads were quantified with *FeatureCounts* (v.2.0.1) of the *Subread* package (Liao et al., 2014), excluding multi-mapping reads and reads mapping to unannotated regions. The *FeatureCounts* counts matrix was pre-filtered using *DESeq2* by removing genes with fewer than 10 reads in 6 individuals, as per the recommendations of the package developer (Love et al., 2014). In total, approximately 3.84 billion raw reads were found among the 24 samples analysed (Table S1). An average of 39.19 million reads (±3.59 million reads) were quantified per sample, for a total of 940 million (Table S2).

Wald tests were applied, using the DESeq() function, to evaluate differential gene expression (*P*<0.05; log_2_ fold-change >2, <−2) resulting from population, treatment and interaction comparisons. Comparisons for the Wald test were performed via the maximum likelihood estimators (MLEs) of normalized log_2_ average read counts for each of the groups using adjusted *P*-values using the Benjamini–Hochberg method. A principal components analysis (PCA) was performed on the regularized-logarithm-transformed (*rlog*) (Love et al., 2014) read counts matrix with the plotPCA() function (Huber et al., 2015). We also generated an UpSet plot (Lex et al., 2014) for differentially expressed genes using the UpSet() function innate to the *ComplexHeatmap* package v.2.22 (Gu et al., 2016), which uses code found in the *UpSetR* package (Conway et al., 2017).

The *goseq* package (v.3.19) was used to perform a gene ontology (GO) enrichment analysis. We first added gene lengths and annotations manually, which were then used to create a probability weighted function (PWF) using nullp() to determine the base probability of a gene to be differentially expressed based on its length alone. The goseq() command was then used to perform GO enrichment analyses using information in the gene association file (GAF) linked to the *G. aculeatus* reference genome. Only the ontologies present in at least two differentially expressed genes were kept. For the effects of treatment (control versus alkaline) within each population, GO terms were further simplified and summarized using REVIGO (http://revigo.irb.hr/; Supek et al., 2011) and semantic similarities between collapsed GO terms were calculated using SimRel (Schlicker et al., 2006) through the REVIGO platform.

### Statistics

Data for survival and physiological parameters are presented as means±s.e.m. All statistical analyses and plotting were performed using R programming language (v.4.3.2) in RStudio (2023.12.1, Build 402; https://www.r-project.org/) and significance was accepted at the *P<*0.05 level. Statistical significance was assessed using generalized linear models (GLMs) [glm() function; https://www.r-project.org/] with mass, population (i.e. lake origin), time in alkaline water (0=control), and the interaction between population and time as factors. Normality and heteroscedasticity were assessed visually using normal *Q–Q* plots and residual versus fitted values plots, respectively. Data were transformed (specific transformations are described in figure captions) if the model failed these qualitative visual inspections. The effects of mass, population, time in alkaline water and the interaction between population and time were assessed using an ANOVA [aov() function; https://www.r-project.org/]. *P*-values for each term and descriptions of data transformations are presented in corresponding figure captions. Estimated marginal means [emmeans() function; https://CRAN.R-project.org/package=emmeans] with a Tukey adjustment for multiple comparisons were used for *post hoc* analyses.

## RESULTS

**Fig. 1.**
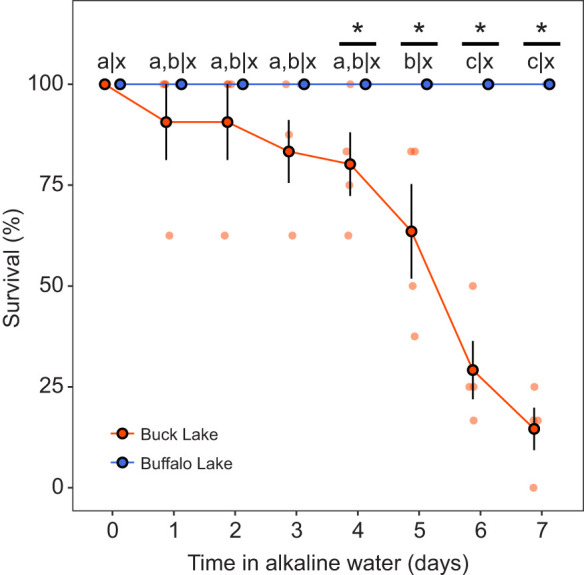
**Survival of brook stickleback collected from pH-neutral Buck Lake or the highly alkaline Buffalo Lake and exposed to alkaline water for 7 days.** Survival data are means±s.e.m. with individual data points presented as transparent symbols. Asterisks represent time points at which there is a statistically significant effect of population. Time points that do not share the same lowercase letter (a,b,c for Buck Lake; x,y,z for Buffalo Lake) are significantly different from one another (*P*_lake_<0.001, *P*_time_<0.001, *P*_lake×time_<0.001).

Survival of Buck Lake and Buffalo Lake stickleback in holding conditions was high (>95%) and survival over a 7 day period in control facility water was 100% (control survival experiment with a single replicate tank of 8 fish for each population; data not shown). Under alkaline conditions, Buffalo Lake stickleback showed 100% survival over a 7 day period, whereas survival in Buck Lake fish was significantly lower than that of Buffalo Lake fish by 4 days and declined significantly from the 0 day 100% survival value starting at 5 days of exposure (Fig. 1). At the termination of the 7 day exposure period, survival was only 14.6±5.2% in Buck Lake stickleback (Fig. 1).

**Fig. 2.**
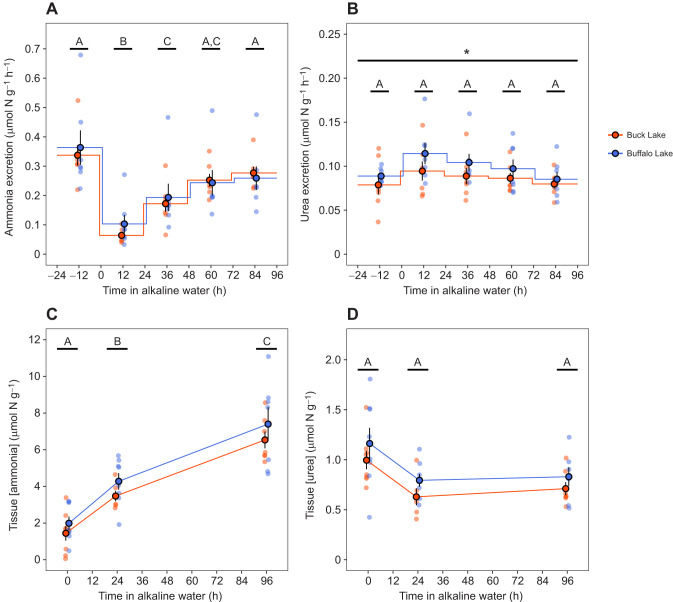
**Nitrogen regulation in brook stickleback collected from Buck Lake or Brook Lake and exposed to alkaline water for 96 h.** Ammonia-N (A) and urea-N (B) excretion rates and tissue [ammonia-N] (C) and [urea-N] (D). Data are means±s.e.m. with individual data points presented as transparent symbols. A single asterisk represents an overall statistically significant effect of population. Time points that do not share the same uppercase letter (A,B,C for overall effect of time) are significantly different from one another (A: log-transformed: *P*_mass_=0.399, *P*_lake_=0.870, *P*_time_<0.001, *P*_lake×time_=0.647; B: log-transformed: *P*_mass_=0.449, *P*_lake_=0.035, *P*_time_=0.116, *P*_lake×time_=0.933; C: square root-transformed: *P*_mass_=0.005, *P*_lake_=0.941, *P*_time_<0.001, *P*_lake×time_=0.931; D: log-transformed: *P*_mass_=0.702, *P*_lake_=0.046, *P*_time_=0.064, *P*_lake×time_=0.865).

Ammonia excretion rates were not affected by population, and exposure to alkaline water resulted in an initial 81% inhibition in Buck Lake stickleback and 72% inhibition in Buffalo Lake stickleback from 0 to 24 h of exposure (Fig. 2A). By 48–72 h of exposure, ammonia excretion rates were not significantly different from pre-exposure rates, indicating that fish in both populations had recovered from the initial inhibition observed over the first 48 h of exposure (Fig. 2A). Urea excretion was significantly greater in Buffalo Lake stickleback compared with Buck Lake stickleback, demonstrating an average 13% higher excretion rate throughout the entire measurement period, but urea excretion was unaffected by the alkaline treatment (Fig. 2B). Tissue [ammonia] in carcass samples was not significantly different between Buffalo and Buck Lake fish and increased significantly by approximately 4-fold over the course of the 96 h exposure to alkaline water (Fig. 2C). Although our model indicated a statistically significant effect of population on tissue [urea] (*P*_lake_=0.046), this effect was not significant in our *post hoc* analysis, and exposure to alkaline water similarly did not affect [urea] in the carcass of fish from either population. (Fig. 2D).

**Fig. 3.**
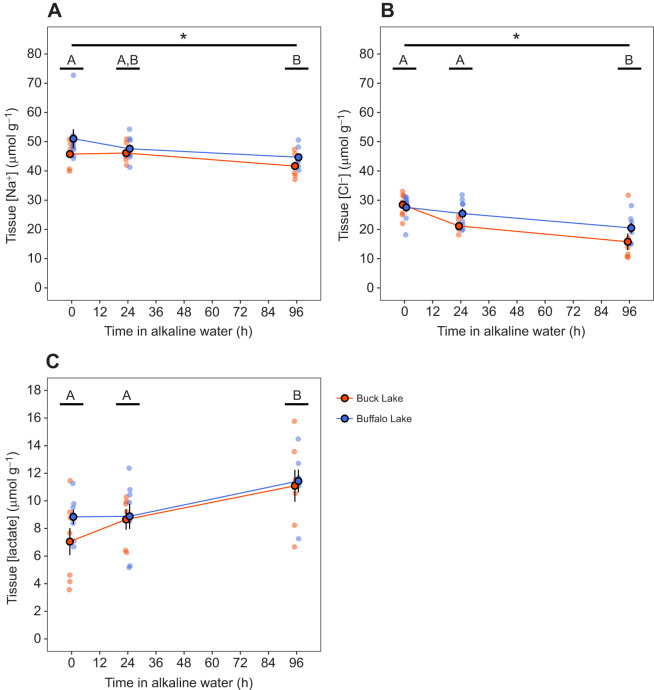
**Ion regulation and tissue lactate concentration in brook stickleback collected from Buck Lake or Buffalo Lake and exposed to alkaline water for 96 h.** Tissue [Na^+^] (A), [Cl^−^] (B) and [lactate] (C) in stickleback carcass samples. Data are means±s.e.m. with individual data points presented as transparent symbols. Asterisks represent time points at which there is a statistically significant effect of population. Time points that do not share the same uppercase letter (A,B,C for overall effect of time) are significantly different from one another; no significant interaction terms were detected for the effect of lake (population) and time (A: *P*_mass_=0.583, *P*_lake_=0.030, *P*_time_=0.034, *P*_lake×time_=0.562; B: *P*_mass_=0.436, *P*_lake_=0.010, *P*_time_<0.001, *P*_lake×time_=0.311; C: *P*_mass_=0.327, *P*_lake_=0.540, *P*_time_=0.002, *P*_lake×time_=0.640).

**Fig. 4.**
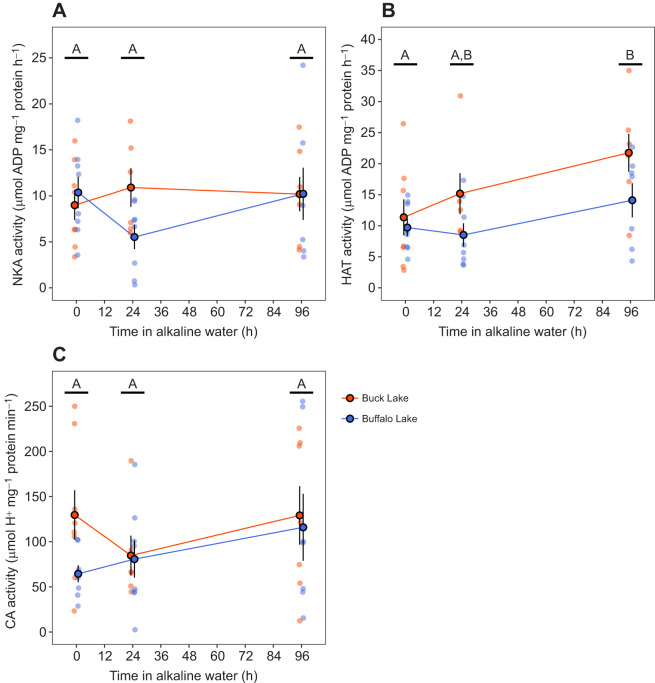
**Enzyme activity in gill tissue of brook stickleback collected from Buck Lake or Buffalo Lake and exposed to alkaline water for 96 h.** Activity of Na^+^/K^+^-ATPase (NKA; A), H^+^-ATPase (HAT; B) and carbonic anhydrase (CA; C). Data are means±s.e.m. with individual data points presented as transparent symbols. Time points that do not share the same uppercase letter (A,B,C for overall effect of time) are significantly different from one another (A: *P*_mass_=0.088, *P*_lake_=0.823, *P*_time_=0.744, *P*_lake×time_=0.174; B: *P*_mass_=0.001, *P*_lake_=0.597, *P*_time_=0.001, *P*_lake×time_=0.108; C: *P*_mass_=0.019, *P*_lake_=0.937, *P*_time_=0.4101, *P*_lake×time_=0.549).

Carcass [Na^+^] was significantly higher (7% on average) in Buffalo Lake fish compared with Buck Lake fish across the entire experimental period (Fig. 3A). Exposure to alkaline water resulted in an overall significant reduction in carcass [Na^+^] by 96 h of exposure, with Buck Lake and Buffalo Lake stickleback demonstrating 9% and 12% reductions, respectively (Fig. 3A). Tissue [Cl^−^] demonstrated similar trends to [Na^+^], with Buffalo Lake fish having significantly higher levels (12% higher on average). However, the impact of alkaline exposure on carcass [Cl^−^] was much greater compared with [Na^+^], such that concentrations were reduced by 44% and 25% in Buck Lake and Buffalo Lake stickleback, respectively, by 96 h of exposure (Fig. 3B). Tissue lactate increased significantly by approximately 50% in both populations by 96 h of exposure to alkaline water, but showed no effects of population (Fig. 3C). No effects of population were observed for gill Na^+^/K^+^-ATPase, H^+^-ATPase or carbonic anhydrase activity, but H^+^-ATPase activity increased overall by 92% and 45% in Buck Lake and Buffalo Lake stickleback, respectively, from 0 to 96 h of alkaline exposure (Fig. 4).

**Fig. 5.**
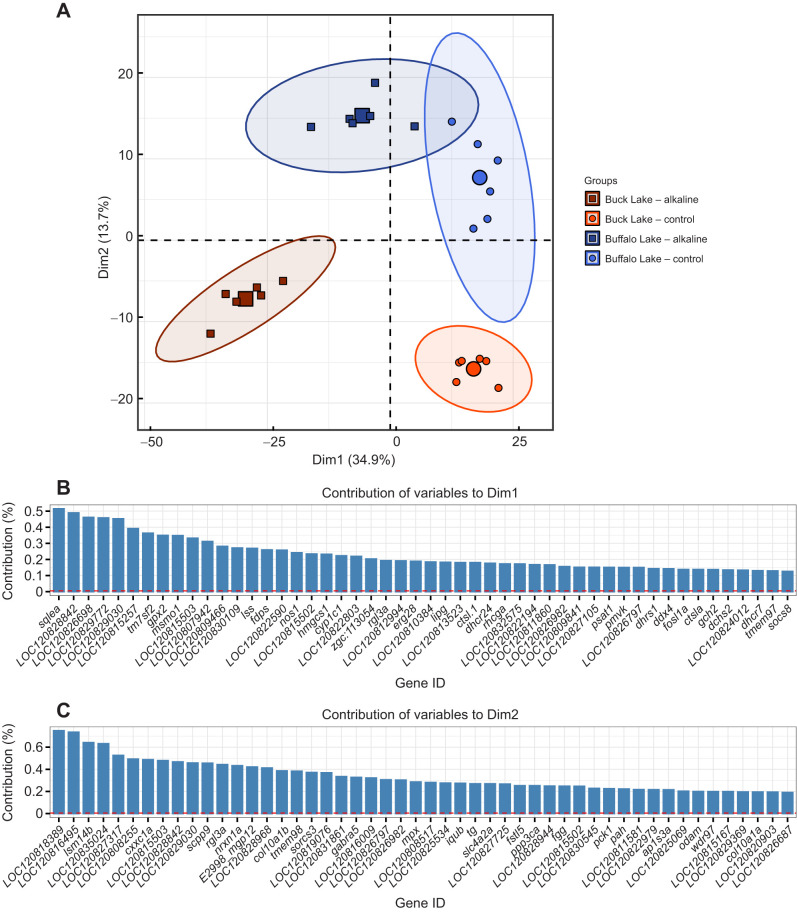
**Gene expression in gill tissue of brook stickleback collected from Buck Lake or Buffalo Lake and exposed to alkaline water for 96 h.** Principal component analysis (PCA) of genes expressed in gill tissue of sticklebacks exposed to control (circles; lighter colours) or alkaline (squares; darker colours) water (A). Larger symbols represent the centroids of the 95% confidence interval ellipses for each group. PCA dimension 1 (Dim1) accounted for 34.9% of the variation in gene expression and PCA dimension 2 (Dim2) accounted for 13.7% of variation in gene expression. The 30 genes with the largest contributions to Dim1 (B) and Dim2 (C) of the PCA are also presented.

Differences in gene expression (i.e. changes in transcript abundance) between populations and treatments were first examined by PCA (Fig. 5). PCA dimension 1 (Dim1) accounted for 34.9% of the variation in gene expression among fish in our study, and fish exposed to alkaline water appeared to increase along Dim1 in principal component space. Notably, the change in Dim1 appeared much greater for Buck Lake stickleback compared with Buffalo Lake stickleback. PCA dimension 2 (Dim2) accounted for 13.7% of the variation and clearly delineated the two populations. Many of the genes contributing substantially (>0.25%) to Dim1 (Fig. 5B) were related to steroid biosynthesis (squalene epoxidase a, *sqlea*; EBP cholestenol delta-isomerase, *LOC120829772*; 3-hydroxy-3-methylglutaryl-coenzyme A reductase-like, *LOC120815257*; methylsterol monooxygenase 1, *msmo1*; lanosterol synthase, *lss*; cytochrome P450 family 51, *LOC120826698*) with most of these genes being upregulated in both populations, but to a greater extent in Buck Lake fish (Table S3). Dim1 was also influenced by at least one gene related to oxidative stress (glutathione peroxidase 2, *gpx2*), which was also more highly upregulated in Buck Lake stickleback. The genes contributing to Dim2 (Fig. 5C) conversely showed less consistent patterns in terms of conserved functions, though notably *slc4a2a*, a bicarbonate transporter involved in intracellular pH regulation, contributed to this PCA dimension. Other notable genes contributing to PCA Dim2 were protein lifeguard 1-like (*LOC120816495*) which plays a role in apoptosis and several genes related to cellular signalling (ral guanine nucleotide dissociation stimulator-like 3a, *rgl3a*; calcium/calmodulin-dependent 3′,5′-cyclic nucleotide phosphodiesterase 1A-like, *LOC120816009*; adhesion G protein-coupled receptor F5-like, *LOC120808517*; and protein phosphatase 3, catalytic subunit, alpha isozyme, *ppp3ca*).

**Fig. 6.**
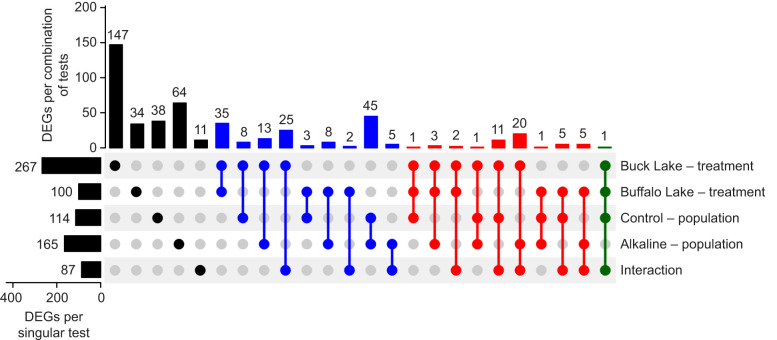
**UpSet plot of genes in gill tissue of brook stickleback that were differentially expressed by exposure to alkaline water within a given population (Buck Lake/Buffalo Lake – treatment), differentially regulated between populations within a given treatment (control/alkaline – population), or differentially regulated as a result of interaction between population and treatment terms.** The *y*-axis represents the number of differentially expressed genes (DEGs) for a given comparison of treatment or population effects and their interaction. The *x*-axis represents the number of DEGs in different combinations of the five comparisons listed on the *y*-axis.

**Fig. 7.**
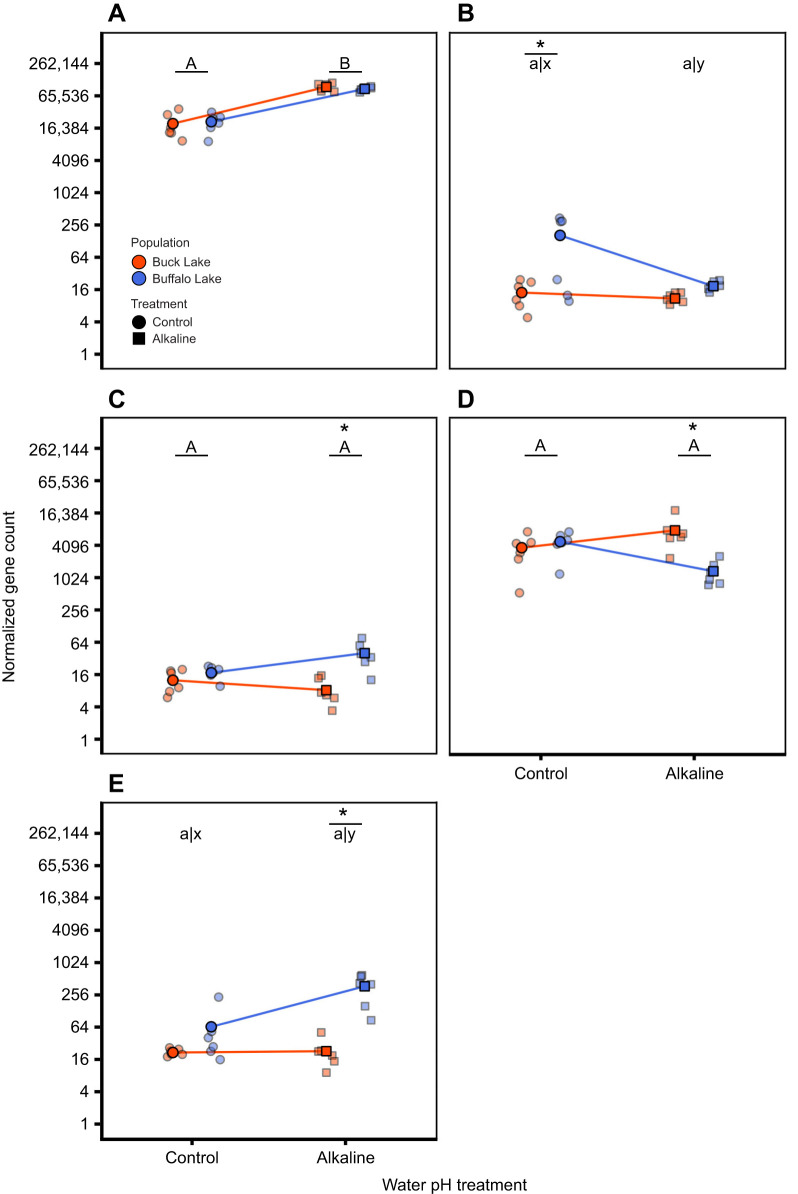
**Expression of genes coding for proteins with putative roles in nitrogen, ion and/or acid–base regulation in gill tissue of brook stickleback collected from Buck Lake or Buffalo Lake and exposed to control or alkaline water for 96 h.** Data are mean (opaque symbols) and raw (transparent symbols) log_2_ fold-counts for *rhcga* (Rh family, C glycoprotein A; A), *LOC120815849* (V-type ATPase subunit d 1-like; B), *ca12* (carbonic anhydrase XII; C), *ca15b* (carbonic anhydrase XVb; D) and *slc4a2a* (solute carrier family 4 member 2a; E). Asterisks represent a statistically significant effect of population within control or alkaline treatments. Means within a population that do not share the same lowercase letter (a,b for Buck Lake; x,y for Buffalo Lake) represent a population-specific, statistically significant effect of alkaline treatment in the case of a significant interaction term. In the case of a non-significant interaction term, means that do not share the same uppercase letter (A,B) represent a population-independent, statistically significant effect of alkaline treatment (*P*<0.001 in all cases of significance; see Table S3 for adjusted *P*-values).

The general trend of a greater transcriptional response to alkaline exposure in Buck Lake stickleback compared with Buffalo Lake stickleback observed by PCA was corroborated by the results presented in the UpSet plot (Fig. 6), which depicts genes that were differentially expressed (488 total) in population and treatment comparisons and their interaction. The population and treatment combination that resulted in the largest number of differentially expressed genes was the effect of alkaline treatment in Buck Lake stickleback (267 genes total). Among these genes that were differentially expressed in Buck Lake stickleback in response to alkaline treatment, the majority were uniquely regulated by this treatment (147) or showed a population interaction (25). Only a small subset of these genes (35) were differentially expressed in both populations and additionally showed an interactive effect between populations (2). In contrast to Buck Lake fish, far fewer genes (100) showed differential regulation in response to alkaline treatment in Buffalo Lake stickleback. Similarly, fewer genes were differentially regulated between populations under control conditions (114 genes) compared with alkaline conditions (165 genes). Among those genes that were differentially expressed between populations, a total of 45 were differentially expressed irrespective of treatment. The complete list of differentially expressed genes is given in Table S3. Some notable differentially expressed genes code for proteins with putative roles in nitrogen, ion and acid–base balance, including Rh family, C glycoprotein A (*rhcga*), V-type ATPase subunit d 1-like (*LOC120815849*), carbonic anhydrase XII (*ca12*), carbonic anhydrase XVb (*ca15b*) and solute carrier family 4 member 2a (*slc4a2a*) (Fig. 7).

**Fig. 8.**
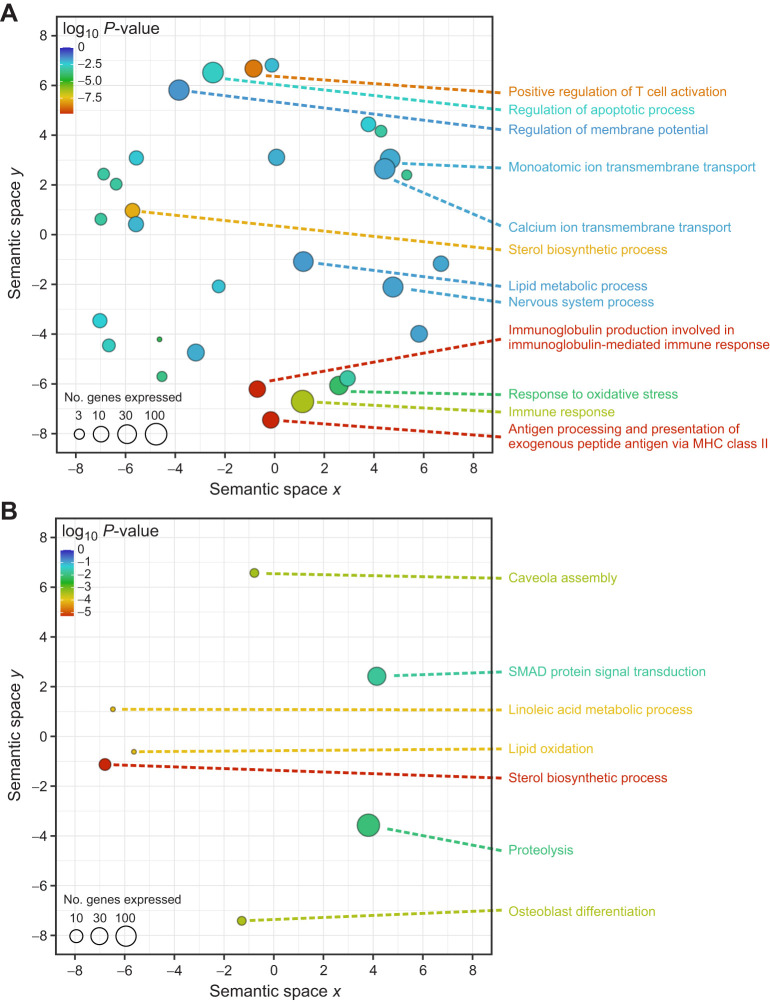
**Scatterplots demonstrating the semantic similarities of over-represented biological processes gene ontology (GO) terms for genes that were differentially expressed between control and alkaline treatment groups from the two lakes.** Data are shown for Buck Lake (A) and Buffalo Lake (B) stickleback.

GO enrichment analysis (Fig. 8; Tables S4–S7) revealed relatively few terms related specifically to membrane transport functions (i.e. pathways that might contribute to branchial regulation of ion, acid–base and/or nitrogen balance) that were over-expressed in our population and treatment comparisons. However, we did observe that the term ‘membrane’ was the most over-represented GO term between populations under alkaline conditions (Table S7). Moreover, in response to alkaline exposure, Buck Lake stickleback showed significant enrichment of several GO terms related to cellular stress and immune responses (Fig. 8A; Table S4) including ‘positive regulation of T cell activation’, ‘regulation of apoptotic process’, ‘immunoglobulin production involved in immunoglobulin-mediated immune response’, ‘response to oxidative stress’, ‘immune response’ and ‘antigen processing and presentation of exogenous peptide antigen via MHC class II’. In contrast, Buffalo Lake stickleback showed far fewer differentially regulated biological processes in response to alkaline treatment and no enrichment of GO terms specifically related to stress or immune responses under alkaline conditions (Fig. 8B; Table S5).

## DISCUSSION

The mechanisms used by fishes to tolerate and thrive in alkaline environments have been investigated in only a small number of highly specialized species, despite many alkaline lakes supporting diverse fish populations (Mitchell and Prepas, 1990; Wood, 2022). Therefore, we sought to characterize mechanisms of alkaline tolerance in brook stickleback inhabiting the alkaline Buffalo Lake in Alberta, Canada. In our study, fish were exposed to high pH and alkalinity to match the water chemistry of Buffalo Lake (Table 1). It is notable, however, that physiological responses to elevated pH can vary depending on water chemistry conditions such as hardness (Wilson et al., 1998) and high ambient [HCO_3_^−^]. Specifically, [HCO_3_^−^] may alter ionoregulatory and acid–base responses to high pH water (see ‘Ion and acid–base regulation’, below).

We initially hypothesized that brook stickleback from Buffalo Lake would have greater alkaline tolerance than those from a reference lake (Buck Lake), and that this would manifest as an increased capacity to regulate physiological processes that are typically impacted by exposure to alkaline or high pH water. Despite a 2 month acclimation to neutral holding conditions, Buffalo Lake stickleback showed no mortality when exposed to alkaline water of similar composition to Buffalo Lake (pH 9.5) for a period of up to 7 days. In contrast, Buck Lake fish showed reduced survival during exposure to alkaline water, demonstrating clear intraspecific differences in alkaline tolerance between these populations. Intraspecific differences in alkaline tolerance have been examined previously in populations of roach (*Rutilus rutilus*), rudd (*Scardinius erythrophthalamus*) and European perch (*Perca fluviatilis*) inhabiting eutrophic and non-eutrophic lakes, the former experiencing regular periods of high alkalinity during summer algal blooms (Scott and Wilson, 2007). Exposure to pH 9.5 in the lab resulted in characteristic disruptions of ammonia and sodium handling in all three species; however, no intraspecific differences between eutrophic and non-eutrophic populations were observed. In contrast, evidence supporting intraspecific differences in alkaline tolerance was observed in Magadi tilapia (*A. grahami*) populations that are separated into isolated lagoons by the presence of a sodium carbonate crust (‘trona’) that overlies the lake and theoretically prevents gene flow between lagoons (Wilson et al., 2004). Despite evidence of minimal population structure among populations from mitochondrial DNA analyses, individuals from the different lagoon populations displayed distinct physiological characteristics that matched the chemical conditions of their respective lagoons, including tolerance to elevated pH and carbonate alkalinity, representing intraspecific differences in alkaline tolerance in this unique species (Wilson et al., 2004). Population-specific differences in tolerance to environmental pH have also been demonstrated in the context of acidic environments. Yellow perch (*Perca flavescens*) collected from naturally acidic lakes (pH 4.5–4.6) survived exposure to pH 3.2 in lab experiments for a significantly longer duration than individuals collected from neutral lakes (pH 7.6–7.8); however, these differences in tolerance were not a function of sodium regulation (Lyons, 1982), the loss of which is believed to be the lethal mechanism of action of acid exposure in freshwater fishes (McDonald, 1983).

By sampling both populations at 4 days of exposure, prior to the observed decrease in survivorship in Buck Lake stickleback (Fig. 1), we aimed to reveal the physiological basis of alkaline tolerance. Contrary to our hypothesis, we were able to detect only subtle differences in the physiological responses to alkaline exposure between the two populations, with a trend towards reduced Cl^−^ regulation and the implication of altered acid–base status being the only potential intraspecific difference that we observed. In contrast, at the level of the transcriptome, there were very clear population-specific differences in gene expression patterns in the gill. Even under neutral control conditions, following 2 months of acclimation to common laboratory conditions, we observed a clear difference in the overall pattern of gene expression between populations that was largely associated with PCA Dim2 (13.7% of the total variation). Moreover, a much larger number of genes was differentially expressed in response to alkaline exposure in Buck Lake fish compared with those from Buffalo Lake. In Buck Lake stickleback, many of these genes were related to cellular stress and immune responses, highlighting the fact that exposure to alkaline water was clearly much more stressful in this population compared with Buffalo Lake stickleback. Overall, these findings highlight marked intraspecific differences in alkaline tolerance that result from habitation of a naturally alkaline lake.

### Nitrogen regulation

Based on our initial hypothesis that Buffalo Lake stickleback would have increased alkaline tolerance due to the high pH of their environment (Table 1), we predicted that they would show different patterns of nitrogen regulation in response to alkaline exposure compared with Buck Lake stickleback. Contrary to our hypothesis, both populations exhibited an initial inhibition of ammonia excretion rates upon exposure to alkaline water, followed by a recovery by 96 h of exposure (Fig. 2A), and there were no differences in carcass tissue [ammonia] between the populations (Fig. 2C). Notably, tissue [ammonia] was relatively high in our study, potentially due to sampling time and because tissues were not freeze clamped; however, the values we report are still within the range observed in rainbow trout white muscle using a variety of sampling and analytical approaches (Wang et al., 1994a). The observed pattern of ammonia excretion inhibition followed by a subsequent full or partial recovery has been demonstrated in many fish species exposed to alkaline conditions (Kumai et al., 2015; Scott et al., 2005; Wilkie and Wood, 1991; Wilkie et al., 1993, 1994; Wright et al., 1993). Recovery of ammonia excretion rates is likely a function of: (1) the accumulation of ammonia in the blood/tissues (Wilkie and Wood, 1991; Wilkie et al., 1993, 1994; Wright et al., 1993; Zhao et al., 2024) which, coupled to a blood alkalosis (see Introduction), presumably restores an outwardly directed *P*_NH_3__ gradient; (2) changes in the function of branchial ionocytes (Laurent et al., 2000; Wilkie and Wood, 1994; Wilkie et al., 1994); and/or (3) increased expression of Rhesus (Rh) glycoproteins (Kumai et al., 2015; Sashaw et al., 2010; Zhao et al., 2024), which are ammonia channels that are critically important to the ammonia excretion mechanism of most fish species (Wright and Wood, 2009; Zimmer, 2024). In our transcriptomic analyses, the expression of one Rh gene, *rhcga*, was significantly increased in response to alkaline treatment in both populations (Fig. 7A), but there were no significant differences between populations, indicating a potential for Rh-mediated ammonia excretion under alkaline conditions in this species. In zebrafish, *rhcga* (formerly *rhcg3*) is not expressed in gill tissue (Nakada et al., 2007), which could indicate species-specific differences in Rh gene expression. In a comparison of genome-wide variation in single nucleotide polymorphisms (SNPs) between neutral and alkaline fish populations, non-synonymous SNPs in the coding region of *rhcga* were identified that appeared to be convergent across multiple species inhabiting alkaline environments (Zhou et al., 2023). Whether these same mutations exist in Buffalo Lake stickleback or other species, and the functional significance of these mutations in the context of alkaline physiology, present interesting avenues for future research.

Among the physiological consequences of inhabiting high pH environments, the inhibition of ammonia excretion and ensuing accumulation of ammonia in the blood and tissues appears to be the most challenging (Wilkie and Wood, 1996; Wood, 2022). In the present study, carcass [ammonia] increased by 4-fold in response to alkaline exposure in both stickleback populations in our study (Fig. 2C). The challenge of ammonia accumulation in alkaline water is perhaps best exemplified by the obligatory ureotelic Magadi tilapia, which expresses a functional OUC that acts to convert ammonia to urea and therefore prevent ammonia toxicity (Randall et al., 1989; Wilkie and Wood, 1996; Wood, 2022; Wood et al., 1989). Brook stickleback collected from Buffalo Lake demonstrated an overall increase in urea excretion rate, but no change in tissue [urea] (Fig. 2B,D), independent of alkaline exposure. The proportion of nitrogen excreted as urea, however, was not statistically different between Buck Lake and Buffalo Lake stickleback under alkaline conditions (23.4±0.2% and 25.7±0.8%, respectively, by 84–96 h of exposure). Therefore, it is unlikely that increased urea excretion in Buffalo Lake stickleback contributes substantially to a reduction in the proportion of nitrogenous waste that must be excreted as ammonia. Increased urea excretion rates, and/or accumulation of urea in the plasma and tissues, have also been observed in other species from less extreme alkaline environments (McGeer et al., 1994; Wilkie et al., 1993; Wright et al., 1993; Zhao et al., 2024). However, in these species, urea production is generally believed to be a function of uricolysis due to the lack of a functional OUC (McGeer et al., 1994; Wilkie et al., 1993; Wright et al., 1993). Urea production via uricolysis is also likely to be the case for Buffalo Lake stickleback.

Many alkaline resident species, including the Lahontan cutthroat trout (*Oncorhynchus clarkii henshawi*) from Pyramid Lake, show lower rates of ammonia production relative to fish living in more pH-neutral waters (McGeer et al., 1994; Wilkie et al., 1993; Wright et al., 1993), a trait that has been implicated in their tolerance to alkaline conditions. However, Buffalo Lake stickleback exhibited rates of ammonia excretion and whole-body ammonia levels that were not significantly different from those observed in Buck Lake fish (Fig. 2A,C). Therefore, we found little evidence to suggest that differences in nitrogen regulation contribute to the increased alkaline tolerance of Buffalo Lake stickleback. However, given that exposure to alkaline water resulted in a 4-fold increase in carcass [ammonia] in both populations, it is possible that Buffalo Lake stickleback may be more tolerant of elevated ammonia levels, as suggested by decreased cellular immune and stress responses that were observed at the transcriptomic level (see ‘Transcriptomic stress and immune responses’, below). It is not clear, however, whether a 4-fold increase in tissue [ammonia] would be lethal in brook stickleback. In rainbow trout, plasma [ammonia] increased 6-fold prior to mortality in response to exposure to pH 10.1 in soft water (Yesaki and Iwama, 1992) and >4-fold increases in extracellular fluid [ammonia] have been observed following non-lethal exhaustive exercise in trout (Wang et al., 1994b).

In future studies on alkaline tolerance of fishes indigenous to alkaline lakes, examination of ammonia and urea concentrations and gene expression patterns in brain tissue may provide further insight into the contribution of ammonia tolerance to overall alkaline tolerance. Ammonia is neurotoxic and has been demonstrated to exert excitotoxicity (Wilkie et al., 2011), oxidative stress (Lisser et al., 2017), and disruptions in water balance (Wilkie et al., 2015) in brain tissue. Exposure to alkaline water in common carp (*Cyprinus carpio*) resulted in cellular damage, changes in the activity of antioxidant enzymes, and differential regulation of genes involved in neuroactive ligand–receptor interactions in brain tissue (Shang et al., 2026). Analysis of these endpoints in Buffalo Lake resident fish species may therefore shed more light on mechanisms of alkaline tolerance. Furthermore, *A. tarichi* indigenous to Lake Van (pH 9.8) showed high levels of the ammonia detoxifying enzyme glutamine synthetase in brain tissue (Danulat and Kempe, 1992), which also might be an important aspect of ammonia tolerance in alkaline lake resident species.

### Ion and acid–base regulation

In both populations of brook stickleback, carcass tissue [Na^+^] and [Cl^−^] decreased over the duration of alkaline exposure, similar to the response observed in other species (Wilkie and Wood, 1996; Wood, 2022). However, this response did not differ between populations despite Buffalo Lake stickleback demonstrating overall higher tissue Na^+^ and Cl^−^ levels (Fig. 3A,B). Previous studies have attributed reductions in blood plasma and tissue [Na^+^] and [Cl^−^] to the inhibition of active ion uptake rates, with comparatively smaller effects on ion efflux rates (Scott et al., 2005; Wilkie et al., 1996, 1999). The mechanism of this inhibition has not been fully investigated, but has been attributed to a reduction in H^+^ and HCO_3_^−^ available for Na^+^/H^+^ and Cl^−^/HCO_3_^−^ exchange in response to alkaline/high pH-induced respiratory alkalosis (Wilkie and Wood, 1991; Wilkie et al., 1999; Wood, 2022). In waters with high [HCO_3_^−^], such as Buffalo Lake and the alkaline water used in our study (Table 1), Cl^−^ loss may additionally represent a direct inhibition of Cl^−^/HCO_3_^−^ exchange or a reversal of Cl^−^/HCO_3_^−^ exchange such that Cl^−^ is excreted.

Interestingly, Buck Lake and Buffalo Lake stickleback demonstrated a larger, though non-significant, decrease in tissue [Cl^−^] compared with the reduction in tissue [Na^+^], which might be reflective of an acid–base disturbance as this response indicates an increase in the strong ion difference (SID; see Wood, 2022). In rainbow trout exposed to buffered water at pH 10.5, which was lethal by 3 days of exposure, trout experienced greater decreases in plasma [Cl^−^] compared with decreases in plasma [Na^+^] (i.e. increased SID), and a concomitant increase in [HCO_3_^−^] and pH (McGeer and Eddy, 1998) as would be predicted by SID theory. In contrast, in the alkaline-adapted Lahontan cutthroat trout, exposure to pH 10 in buffered Pyramid Lake water resulted in equal reductions of plasma [Na^+^] and [Cl^−^], such that SID was likely unchanged (Wilkie et al., 1993). Buck Lake stickleback experienced larger decreases in tissue [Cl^−^] than did Buffalo Lake stickleback, which could indicate a greater ionic/acid–base disruption in this population, though this difference was not statistically significant. Although we were unable to directly assess acid–base status of stickleback exposed to alkaline water, the increase in carcass tissue [lactate], which has been observed in response to exposure to alkaline or high pH water in several species (Kwan et al., 2024; Wilkie and Wood, 1991; Wilkie et al., 1993; Wilson et al., 1998; Zimmer et al., 2024), would suggest an increase in H^+^ production through increased glycolytic flux coupled to ATP hydrolysis (Hochachka and Mommsen, 1983). This metabolic H^+^ production would be necessary to counteract a blood alkalosis in response to alkaline water. Similar to tissue [ammonia], tissue [lactate] reported in our study is generally high compared with previously reported values for fish white muscle (Wang et al., 1994a), potentially owing to sampling time or tissue processing. However, the overall trends reflect the typical response to high pH exposure in fish.

GO enrichment analysis revealed that under alkaline conditions, the most over-expressed term between populations was ‘membrane’ (Table S7), a term that was not significantly different under control conditions (Table S6). This treatment-specific difference between populations might indicate that membrane reorganization or other membrane processes (e.g. increased ionocyte density) underlie some of the differences in alkaline tolerance, as has been demonstrated in the Lahontan cutthroat trout (Wilkie et al., 1994). Similarly, both populations demonstrated a significant regulation of genes involved in sterol biosynthesis (Fig. 8), which also might implicate changes in membrane structure in the response to alkaline exposure given the role of cholesterol in regulating membrane structure and function (Cui et al., 2025). Moreover, branchial H^+^-ATPase activity increased in response to alkaline exposure, irrespective of population (Fig. 4B). This response might indicate differences in ionocyte function or density as a result of alkaline exposure, although this would not explain differences in alkaline tolerance between populations. The role of H^+^-ATPase in ion and acid–base balance would also depend on its localization to the apical or basolateral membrane of ionocytes, which is a controversial topic in ionoregulatory physiology (Tseng et al., 2020, 2022). In our study, the observed increase in gill H^+^-ATPase activity would make most sense in the context of basolateral expression which, in conjunction with increased metabolic H^+^ production, would contribute to the correction of a potential alkalosis in response to alkaline exposure. Interestingly, one H^+^-ATPase gene (V-type ATPase subunit d 1-like, *LOC120815849*; Fig. 7B) showed a higher expression in Buffalo Lake fish compared with Buck Lake fish under control conditions, but expression was downregulated in response to alkaline exposure specifically in Buffalo Lake fish. This discrepancy between gene expression and enzyme activity is not clear, but could be related to the fact that multiple genes encode different H^+^-ATPase subunits in fishes, with subunit A playing an important role in H^+^ secretion in zebrafish (Horng et al., 2007).

We also identified a number of additional genes with putative ion and acid–base regulatory functions that were differentially expressed between populations or in response to alkaline treatment. Two carbonic anhydrase genes were differentially expressed in response to alkaline exposure specifically in Buffalo Lake fish. The expression of *ca12* was higher in Buffalo Lake stickleback compared with Buck Lake stickleback under alkaline conditions, while the expression of *ca15b* showed the opposite trend (Fig. 7C,D). At present, the cellular and subcellular localization of these carbonic anhydrase isoforms is not known in brook stickleback and therefore the implications of these results are unclear. In zebrafish, another *ca15* isoform, *ca15a*, is believed to be expressed in the apical membrane of H^+^-ATPase-rich ionocytes (Gilmour and Perry, 2009; Lin et al., 2008), and its branchial expression is increased in response to acidic exposure (Lin et al., 2008). The downregulation of an apical carbonic anhydrase isoform in response to alkaline exposure in Buffalo Lake stickleback would potentially act to retain H^+^ and additionally limit absorption of HCO_3_^−^ from the environment. In Amur ide (*Leuciscus waleckii*), non-synonymous SNPs in *ca15a* and *ca15b* genes were found to be highly differentiated between alkaline and neutral populations (Zhou et al., 2023), suggesting that these genes may play important roles in the adaptation to alkaline environments. The gene encoding anion exchanger 2 (*slc4a2a*), which participates in Cl^−^/HCO_3_^−^ exchange, was upregulated in both stickleback populations in response to alkaline exposure (Fig. 7E), and this upregulation was more pronounced in Buffalo Lake stickleback. Overall, Buffalo Lake fish appeared to mount a greater transcriptional response to alkaline water with respect to these select genes coding for proteins with putative ion and acid–base regulatory functions, which could contribute to the increased tolerance of Buffalo Lake fish to alkaline conditions.

### Transcriptomic stress and immune responses

The results from our transcriptomic analyses clearly demonstrated that Buck Lake fish mounted an overall greater transcriptional response following 96 h of alkaline exposure compared with Buffalo Lake fish (Figs 5A and 6). While we identified few differentially regulated genes that played putative roles in the regulation of nitrogen, ion or acid–base regulation, there were large changes in the expression of genes related to cellular stress and immune responses. The PCA analysis revealed that Dim1, which explained 34.9% of the variation in the PCA and clearly delineated control and alkaline-exposed individuals, comprised several genes related to cholesterol synthesis, oxidative stress and immunity. In both populations, four genes encoding key enzymes in the cholesterol biosynthesis pathway (hydroxy-3-methylglutaryl-coenzyme A reductase-like, *LOC120815257*; squalene epoxidase a, *sqlea*; lanosterol synthase, *lss*; cytochrome P450 family 51, *LOC120826698*) (Cui et al., 2025; Lepesheva and Waterman, 2007; Luo et al., 2020) were upregulated, with this increase being more pronounced in Buck Lake fish (Fig. 8; Table S3). Differential expression of genes involved in sterol biosynthesis in response to exposure to alkaline water has also been demonstrated previously in the gills of Nile tilapia (*Oreochromis niloticus*; Zhao et al., 2020) and liver of Amur ide (Xu et al., 2013), suggesting that this response to alkaline water may be conserved across species. Cholesterol plays several cellular roles including the production of steroid hormones, regulation of membrane fluidity and cellular signalling (Cui et al., 2025), and activation of the cholesterol synthesis pathway has been identified as a general stress response in some cell types (Shack et al., 1999).

The diminished upregulation of cholesterogenic genes in Buffalo Lake stickleback may represent an example of transcriptomic resilience or dampening (Rivera et al., 2021), whereby this population was able to recover from the initial stress of alkaline exposure, or displayed a reduced response overall. This dampened transcriptional response may serve to reduce energy expenditure or prevent the activation of detrimental cellular stress responses. Indeed, Buck Lake fish demonstrated differential expression of pro-apoptotic and oxidative stress response genes, whereas these biological processes were not differentially regulated in Buffalo Lake fish (Fig. 8). The apoptotic genes that were differentially regulated in our analyses, including caspase recruitment domain-containing proteins and NACHT, LRR and PYD domain-containing proteins, were significantly upregulated in response to alkaline exposure only in the Buck Lake population (Table S3). These transcriptomic changes of apoptotic genes are consistent with the observation of gill damage in response to alkaline exposure in other fish species (Huang et al., 2026; Ye et al., 2025; Zhu et al., 2023); however, histopathological effects of alkaline exposure were not assessed in the current study.

GO analyses demonstrated that several pathways related to oxidative stress and immune responses were also enriched in response to alkaline exposure specifically in Buck Lake fish, while these pathways were not significantly enriched in Buffalo Lake fish exposed to alkaline water (Fig. 8). Interestingly, genes with immune response ontologies were both downregulated (e.g. several H-2 class II histocompatibility antigen genes) and upregulated (e.g. tumour necrosis factor a, interleukin 22) (Table S3) in Buck Lake stickleback, highlighting a complex immune response to alkaline exposure in this population. Differential regulation of genes with ontologies related to immune function appears to be another well-conserved response to alkaline water across multiple fish species (Huang et al., 2026; Ye et al., 2025; Zhao et al., 2020; Zhu et al., 2023), suggesting that alkaline conditions may trigger cellular stress and immunological responses. The diminished immune response at the transcriptomic level in Buffalo Lake stickleback may represent another important aspect of increased alkaline tolerance in this population, indicating a dampened cellular stress response, similar to the trends observed in sterol biosynthesis and apoptotic pathways.

### Conclusions and perspectives

Buffalo Lake, Alberta, is one of many fish-supporting alkaline lakes present in arid regions globally. In the present study, we demonstrated that alkaline habitation results in increased alkaline tolerance and that brook stickleback from a more pH-neutral lake (Buck Lake) showed poor survival under alkaline conditions in the laboratory. Contrary to our initial hypothesis, alkaline tolerance was largely independent of physiological differences in nitrogen, ion or acid–base regulation. Exposure to alkaline water in Buffalo Lake stickleback did, however, appear to be associated with a dampened cellular stress response compared with Buck Lake fish. This dampened response could indicate reduced stress and immune responses to alkaline conditions that might confer increased alkaline tolerance in this population. To our knowledge, this study represents one of the first direct comparisons of intraspecific alkaline tolerance between alkaline and neutral populations of a single species. Future work in this area should aim to determine whether these intraspecific differences are a function of evolved tolerance through local adaptation or are driven by developmental plasticity, and the role of other tissues (e.g. liver and brain) in acquired alkaline tolerance in alkaline fish populations.

Regardless of the mechanism underlying alkaline tolerance in Buffalo Lake stickleback, these findings have important implications for our understanding of the ecophysiology of alkaline lake fishes. A framework of ‘chemical niches’ has been previously proposed to better understand the distribution of fishes along chemical gradients in the wild (Zimmer et al., 2021). The case of Buffalo Lake stickleback clearly demonstrates an expansion of the ‘pH niche’ of this species and suggests that some populations may be uniquely adapted to these conditions, a finding that might shape management approaches. Indeed, stocking of alkaline-naive salmonids in alkaline lakes has proven difficult (Thompson et al., 2016) and a large body of work is emerging to better understand the physiological implications of expanding aquaculture to underutilized alkaline environments (e.g. Li et al., 2025; Shang et al., 2026; Zhai et al., 2024; Zhang et al., 2025). Whether the presence of other species in Buffalo Lake and other alkaline lakes represents cases of an expanded pH niche achieved through intraspecific differences in alkaline tolerance, or simply a wide pH niche that allows for alkaline habitation, remains to be determined. Addressing this question will be important for understanding mechanisms underlying alkaline tolerance in fishes and species distribution patterns in alkaline lakes across the globe. Moreover, this research adds to our fundamental understanding of the physiological processes underlying responses to alkaline exposure. Broadening our understanding of these processes might have important implications for predicting the effects of transient alkalinization events such as eutrophication (Scott et al., 2005) and ash deposition from wildfires (Kwan et al., 2024).

## Supplementary Material

10.1242/jexbio.251351_sup1 Supplementary information

Table S2. Read mapping and quantification for all samples of brook stickleback gill tissue used in transcriptomic analyses. Values are the number of reads quantified per gene in each sample.

Table S3. Differential gene expression (log_2_ fold-change) and adjusted p values for lake population and alkaline treatment comparisons. Differential expression was determined for the of alkaline treatment relative to the control treatment within lake populations (Buck_Treatment, Buffalo_Treatment) and the effect of lake population (Buffalo relative to Buck) within treatments (Control_Population, Alkaline_Population), and their interaction.
