# Supplementary material for: Intraspecific differences in tolerance to elevated pH and alkalinity in brook stickleback (Culaea inconstans) inhabiting neutral and alkaline lakes
Source: J Exp Biol. 2026 Jul 9;229(13):jeb251351. doi: 10.1242/jeb.251351 (PMC13380978; doi:10.1242/jeb.251351)
Supplement: Supplementary information [file jexbio-229-251351-s1.pdf]

Table S1. Summary of the sequenced RNA reads' quality data and mapping results

Sample ID	Treatment groups	Raw reads	Trimmed reads	GC %	Primary mapped reads	Total alignments	Quantified alignments
**Culaea_37**	Buck-Control	147,378,696	131,635,626	50-50	83,581,359 (63.49%)	53,754,660	35,663,317 (66.34%)
**Culaea_38**	Buck-Control	187,716,536	167,218,114	50-50	105,624,881 (63.17%)	68,864,100	45,187,844 (65.62%)
**Culaea_39**	Buck-Control	150,911,654	133,794,086	50-50	84,153,362 (62.9%)	54,465,774	36,185,488 (66.44%)
**Culaea_40**	Buck-Control	149,827,886	134,714,274	50-51	85,529,442 (63.49%)	57,828,021	35,782,679 (61.88%)
**Culaea_41**	Buck-Control	156,557,586	137,750,004	50-50	86,760,580 (62.98%)	57,767,770	36,864,539 (63.82%)
**Culaea_42**	Buck-Control	156,255,126	137,899,092	50-51	88,005,470 (63.82%)	56,959,898	37,825,577 (66.41%)
**Culaea_47**	Buffalo-Control	168,792,036	152,186,410	50-50	98,554,049 (64.76%)	62,008,681	42,866,813 (69.13%)
**Culaea_48**	Buffalo-Control	153,129,072	137,198,492	50-50	86,998,732 (63.41%)	56,451,438	37,857,177 (67.06%)
**Culaea_49**	Buffalo-Control	150,777,714	135,801,176	50-50	87,741,697 (64.61%)	55,632,910	37,934,940 (68.19%)
**Culaea_50**	Buffalo-Control	164,366,594	147,930,552	50-51	95,254,837 (64.39%)	73,751,213	38,758,619 (52.55%)
**Culaea_51**	Buffalo-Control	176,731,248	155,258,712	50-50	97,853,056 (63.03%)	64,356,087	41,179,524 (63.99%)
**Culaea_52**	Buffalo-Control	171,301,812	152,633,168	50-51	97,448,544 (63.84%)	63,292,620	41,904,156 (66.21%)
**Culaea_71**	Buck-Alkaline	165,895,346	146,617,620	50-50	92,952,392 (63.4%)	58,669,890	40,994,609 (69.87%)
**Culaea_72**	Buck-Alkaline	166,600,250	147,439,358	50-51	95,176,561 (64.55%)	61,095,450	41,445,540 (67.84%)
**Culaea_73**	Buffalo-Alkaline	134,423,276	119,607,360	50-50	101,476,721 (84.84%)	67,654,874	43,691,405 (64.58%)
**Culaea_74**	Buffalo-Alkaline	172,741,728	155,232,026	50-50	90,590,080 (58.36%)	58,703,221	39,461,488 (67.22%)

Journal of Experimental Biology • Supplementary information

**Culaea_75**	Buffalo-Alkaline	146,509,992	132,234,326	50-51	103,213,336 (78.05%)	66,136,640	45,516,036 (68.82%)
**Culaea_76**	Buffalo-Alkaline	159,387,156	144,312,804	50-51	84,377,368 (58.47%)	54,134,972	37,084,246 (68.5%)
**Culaea_78**	Buck-Alkaline	179,196,796	158,346,728	50-51	76,453,699 (48.28%)	48,278,840	33,813,905 (70.04%)
**Culaea_79**	Buck-Alkaline	159,945,330	141,430,158	50-50	99,276,843 (70.19%)	64,317,370	43,616,691 (67.81%)
**Culaea_81**	Buffalo-Alkaline	179,477,900	161,740,148	50-50	87,042,346 (53.82%)	54,649,094	38,359,987 (70.19%)
**Culaea_82**	Buffalo-Alkaline	150,696,148	132,552,312	50-50	77,307,504 (58.32%)	61,614,212	31,258,261 (50.73%)
**Culaea_84**	Buck-Alkaline	151,743,180	135,533,384	50-50	85,345,841 (62.97%)	55,988,169	36,962,221 (66.02%)
**Culaea_85**	Buck-Alkaline	135,670,734	120,581,292	50-51	93,404,781 (77.46%)	61,531,240	40,454,137 (65.75%)
**Total**		3,836,033,796	3,419,647,222		2,184,123,481 (63.87%)	1,437,907,144	940,669,199 (65.42%)

**Statistics**	**Average (±S.E.)**
**Raw reads**	159,834,742 (±13,747,472)
**Trimmed reads**	142,485,301 (±12,260,729)
**Primary aligned reads**	91,005,145 (±7,824,763)
**Alignments**	59,912,798 (±5,758,106)

Table S2. Read mapping and quantification for all samples of brook stickleback gill tissue used in transcriptomic analyses. Values are the number of reads quantified per gene in each sample.

Available for download at
https://journals.biologists.com/jeb/article-lookup/doi/10.1242/jeb.251351#supplementary-data

Table S3. Differential gene expression (log_2 fold-change) and adjusted p values for lake population and alkaline treatment comparisons. Differential expression was determined for the of alkaline treatment relative to the control treatment within lake populations (Buck_Treatment, Buffalo_Treatment) and the effect of lake population (Buffalo relative to Buck) within treatments (Control_Population, Alkaline_Population), and their interaction.

Available for download at
https://journals.biologists.com/jeb/article-lookup/doi/10.1242/jeb.251351#supplementary-data

Table S4. GO enrichment analysis of genes differentially regulated by alkaline treatment within Buck Lake population

Category	Number of differentiated genes in category	Number of genes in category	GO Term	Gene Ontology	P-value
GO:0002381	8	14	immunoglobulin production involved in immunoglobulin-mediated immune response	BP	1.63725381138553e-10
GO:0002503	8	14	peptide antigen assembly with MHC class II protein complex	BP	1.63725381138553e-10
GO:0019886	8	14	antigen processing and presentation of exogenous peptide antigen via MHC class II	BP	1.63725381138553e-10
GO:0023026	8	14	MHC class II protein complex binding	MF	1.63725381138553e-10
GO:0042613	8	14	MHC class II protein complex	CC	1.63725381138553e-10
GO:0050870	8	18	positive regulation of T cell activation	BP	2.04285728535961e-09
GO:0016126	5	8	sterol biosynthetic process	BP	1.5420778225147e-08
GO:0006695	5	10	cholesterol biosynthetic process	BP	2.51064948399445e-07
GO:0006955	14	154	immune response	BP	3.82669642071242e-07
GO:0008061	5	9	chitin binding	MF	5.24813117034463e-07
GO:0020037	9	100	heme binding	MF	2.53145209280156e-05
GO:0006418	2	2	tRNA aminoacylation for protein translation	BP	4.9661127361181e-05
GO:0016702	3	7	oxidoreductase activity, acting on single donors with incorporation of molecular oxygen, incorporation of two atoms of oxygen	MF	5.44974608589212e-05
GO:0005506	8	91	iron ion binding	MF	5.5297798995395e-05
GO:0016787	5	30	hydrolase activity	MF	8.65023857186677e-05
GO:0006979	5	28	response to oxidative stress	BP	9.64754051613917e-05
GO:0016020	37	1668	membrane	CC	0.000166607

Journal of Experimental Biology • Supplementary information

GO:0038023	6	66	signaling receptor activity	MF	0.000230184
GO:0004100	2	3	chitin synthase activity	MF	0.000269689
GO:0006038	2	3	cell wall chitin biosynthetic process	BP	0.000269689
GO:0030428	2	3	cell septum	CC	0.000269689
GO:0015101	2	3	organic cation transmembrane transporter activity	MF	0.000426074
GO:0015695	2	3	organic cation transport	BP	0.000426074
GO:0003934	2	2	GTP cyclohydrolase I activity	MF	0.000546272
GO:0004812	2	4	aminoacyl-tRNA ligase activity	MF	0.000558614
GO:0004197	5	53	cysteine-type endopeptidase activity	MF	0.000613503
GO:0034440	2	4	lipid oxidation	BP	0.000615687
GO:0043651	2	4	linoleic acid metabolic process	BP	0.000615687
GO:0006642	2	4	triglyceride mobilization	BP	0.000677771
GO:0030301	2	4	cholesterol transport	BP	0.000677771
GO:0034359	2	4	mature chylomicron	CC	0.000677771
GO:0034362	2	4	low-density lipoprotein particle	CC	0.000677771
GO:0042742	3	10	defense response to bacterium	BP	0.000707201
GO:0005219	2	6	ryanodine-sensitive calcium-release channel activity	MF	0.0007294
GO:0005790	2	6	smooth endoplasmic reticulum	CC	0.0007294
GO:0034704	2	6	calcium channel complex	CC	0.0007294
GO:0016262	2	4	protein N-acetylglucosaminyltransferase activity	MF	0.00082117
GO:0004601	4	21	peroxidase activity	MF	0.000838263
GO:0000254	2	3	C-4 methylsterol oxidase activity	MF	0.00102389
GO:0120020	2	5	cholesterol transfer activity	MF	0.001041208
GO:0050750	2	5	low-density lipoprotein particle receptor binding	MF	0.001149677
GO:0005576	12	285	extracellular region	CC	0.001625772
GO:0046085	2	5	adenosine metabolic process	BP	0.001819293
GO:0005811	3	19	lipid droplet	CC	0.001966711
GO:0042981	5	69	regulation of apoptotic process	BP	0.002009542
GO:0034361	2	5	very-low-density lipoprotein particle	CC	0.00204716
GO:0071944	2	7	cell periphery	CC	0.002102864
GO:0033017	2	8	sarcoplasmic reticulum membrane	CC	0.002389365
GO:0106274	2	4	NAD+-protein-arginine ADP-ribosyltransferase activity	MF	0.002765822
GO:1990837	2	7	sequence-specific double-stranded DNA binding	MF	0.003658177
GO:0009117	2	7	nucleotide metabolic process	BP	0.00373316
GO:0042953	2	8	lipoprotein transport	BP	0.00403257
GO:0016746	2	11	acyltransferase activity	MF	0.004464197
GO:0004508	2	7	steroid 17-alpha-monooxygenase activity	MF	0.004753275
GO:0042446	2	7	hormone biosynthetic process	BP	0.004753275
GO:0042448	2	7	progesterone metabolic process	BP	0.004753275
GO:0047442	2	7	17-alpha-hydroxyprogesterone aldolase activity	MF	0.004753275

Journal of Experimental Biology • Supplementary information

GO:0008569	2	15	minus-end-directed microtubule motor activity	MF	0.005334767
GO:0016176	2	9	superoxide-generating NADPH oxidase activator activity	MF	0.005335101
GO:0046654	2	5	tetrahydrofolate biosynthetic process	BP	0.005609396
GO:0004222	5	109	metalloendopeptidase activity	MF	0.006332007
GO:0045663	2	6	positive regulation of myoblast differentiation	BP	0.006918516
GO:0006729	2	6	tetrahydrobiopterin biosynthetic process	BP	0.00809074
GO:0030286	2	16	dynein complex	CC	0.008255916
GO:0008299	2	9	isoprenoid biosynthetic process	BP	0.008360791
GO:0046872	7	206	metal ion binding	MF	0.010084987
GO:0008375	2	12	acetylglucosaminyltransferase activity	MF	0.010143734
GO:0042632	2	13	cholesterol homeostasis	BP	0.010220784
GO:0016485	2	16	protein processing	BP	0.010853302
GO:0034220	3	47	monoatomic ion transmembrane transport	BP	0.011763766
GO:0030674	2	13	protein-macromolecule adaptor activity	MF	0.013373122
GO:0007517	2	10	muscle organ development	BP	0.013596259
GO:0070588	3	62	calcium ion transmembrane transport	BP	0.014533128
GO:0005230	2	18	extracellular ligand-gated monoatomic ion channel activity	MF	0.014795206
GO:0042554	2	15	superoxide anion generation	BP	0.015503359
GO:0050877	3	53	nervous system process	BP	0.01561869
GO:0008253	2	16	5'-nucleotidase activity	MF	0.01789228
GO:0006629	3	46	lipid metabolic process	BP	0.019110773
GO:0005251	2	25	delayed rectifier potassium channel activity	MF	0.019845561
GO:0003950	2	20	NAD+ ADP-ribosyltransferase activity	MF	0.02169218
GO:0008203	2	16	cholesterol metabolic process	BP	0.021972569
GO:0042391	3	62	regulation of membrane potential	BP	0.024128151
GO:0030594	2	24	neurotransmitter receptor activity	MF	0.024128869
GO:0098632	2	30	cell-cell adhesion mediator activity	MF	0.024905537
GO:0051959	2	25	dynein light intermediate chain binding	MF	0.025955518
GO:0016616	2	18	oxidoreductase activity, acting on the CH-OH group of donors, NAD or NADP as acceptor	MF	0.027048765
GO:0009617	3	35	response to bacterium	BP	0.032459263
GO:0005164	2	20	tumor necrosis factor receptor binding	MF	0.034022528
GO:0042383	2	32	sarcolemma	CC	0.039254875
GO:0000166	2	22	nucleotide binding	MF	0.039707136
GO:0050661	2	23	NADP binding	MF	0.039762753
GO:0005615	13	546	extracellular space	CC	0.048003813

Journal of Experimental Biology • Supplementary information

Table S5. GO enrichment analysis of genes differentially regulated by alkaline treatment within Buffalo Lake population

Category	Number of differentiated genes in category	Number of genes in category	GO Term	Gene Ontology	P-value
GO:0008061	5	9	chitin binding	MF	2.62605532400755e-09
GO:0005576	11	285	extracellular region	CC	5.31247697216574e-07
GO:0016126	3	8	sterol biosynthetic process	BP	5.33857904152884e-06
GO:0034440	2	4	lipid oxidation	BP	9.03313953764256e-05
GO:0043651	2	4	linoleic acid metabolic process	BP	9.03313953764256e-05
GO:0004197	4	53	cysteine-type endopeptidase activity	MF	0.00012
GO:0001649	2	5	osteoblast differentiation	BP	0.000331
GO:0016702	2	7	oxidoreductase activity, acting on single donors with incorporation of molecular oxygen, incorporation of two atoms of oxygen	MF	0.000384
GO:0070836	2	5	caveola assembly	BP	0.000409
GO:0005506	4	91	iron ion binding	MF	0.001319
GO:0005901	2	13	caveola	CC	0.002161
GO:0001664	2	27	G protein-coupled receptor binding	MF	0.007047
GO:0006508	5	276	proteolysis	BP	0.009246
GO:0004222	3	109	metalloendopeptidase activity	MF	0.010485
GO:0060090	2	44	molecular adaptor activity	MF	0.015075
GO:0050660	2	40	flavin adenine dinucleotide binding	MF	0.015815
GO:0060395	2	46	SMAD protein signal transduction	BP	0.01625
GO:0005769	2	45	early endosome	CC	0.01637
GO:0005765	2	61	lysosomal membrane	CC	0.03752

Journal of Experimental Biology • Supplementary information

Table S6. GO enrichment analysis of genes differentially regulated between populations within control treatment

Category	Number of differentiated genes in category	Number of genes in category	GO Term	Gene Ontology	P-value
GO:2001234	2	7	negative regulation of apoptotic signaling pathway	BP	0.000547
GO:0016176	2	9	superoxide-generating NADPH oxidase activator activity	MF	0.000838
GO:0004523	2	9	RNA-DNA hybrid ribonuclease activity	MF	0.000886
GO:0007216	2	15	G protein-coupled glutamate receptor signaling pathway	BP	0.002188
GO:0004930	5	188	G protein-coupled receptor activity	MF	0.00233
GO:0042554	2	15	superoxide anion generation	BP	0.002422
GO:0051966	2	16	regulation of synaptic transmission, glutamatergic	BP	0.00249
GO:0005344	2	12	oxygen carrier activity	MF	0.004075
GO:0005833	2	12	hemoglobin complex	CC	0.004075
GO:0031720	2	12	haptoglobin binding	MF	0.004075
GO:0031838	2	12	haptoglobin-hemoglobin complex	CC	0.004075
GO:0043177	2	12	organic acid binding	MF	0.004075
GO:0015671	2	14	oxygen transport	BP	0.005074
GO:0019825	2	15	oxygen binding	MF	0.005533
GO:0072562	2	16	blood microparticle	CC	0.006049
GO:0005254	2	29	chloride channel activity	MF	0.008325
GO:0004601	2	21	peroxidase activity	MF	0.008471
GO:0042744	2	20	hydrogen peroxide catabolic process	BP	0.009835
GO:0007186	5	273	G protein-coupled receptor signaling pathway	BP	0.012393
GO:1902476	2	40	chloride transmembrane transport	BP	0.01527
GO:0020037	3	100	heme binding	MF	0.016835
GO:0008289	2	43	lipid binding	MF	0.019703
GO:0005769	2	45	early endosome	CC	0.020868
GO:0005515	20	2638	protein binding	MF	0.025848
GO:0050877	2	53	nervous system process	BP	0.026335
GO:0070588	2	62	calcium ion transmembrane transport	BP	0.034981
GO:0062023	2	61	collagen-containing extracellular matrix	CC	0.04002

Journal of Experimental Biology • Supplementary information

Table S7. GO enrichment analysis of genes differentially regulated between populations within alkaline treatment

Category	Number of differentiated genes in category	Number of genes in category	GO Term	Gene Ontology	P-value
GO:0016020	24	1668	membrane	CC	0.000321044
GO:0009617	4	35	response to bacterium	BP	0.00035154
GO:0001669	2	4	acrosomal vesicle	CC	0.000382645
GO:0046085	2	5	adenosine metabolic process	BP	0.000407731
GO:0046920	3	17	alpha-(1->3)-fucosyltransferase activity	MF	0.000453304
GO:0036065	3	18	fucosylation	BP	0.000596937
GO:0106274	2	4	NAD+-protein-arginine ADP-ribosyltransferase activity	MF	0.000653164
GO:0009117	2	7	nucleotide metabolic process	BP	0.000851536
GO:2001234	2	7	negative regulation of apoptotic signaling pathway	BP	0.001141344
GO:0038023	4	66	signaling receptor activity	MF	0.001466137
GO:0004523	2	9	RNA-DNA hybrid ribonuclease activity	MF	0.001703961
GO:0016787	3	30	hydrolase activity	MF	0.002008946
GO:0004601	3	21	peroxidase activity	MF	0.002365985
GO:0004089	2	13	carbonate dehydratase activity	MF	0.003117366
GO:0050877	3	53	nervous system process	BP	0.003869661
GO:0008253	2	16	5'-nucleotidase activity	MF	0.004672731
GO:0007268	4	114	chemical synaptic transmission	BP	0.00522694
GO:0004890	2	21	GABA-A receptor activity	MF	0.006547717
GO:1902711	2	21	GABA-A receptor complex	CC	0.006547717
GO:0003950	2	20	NAD+ ADP-ribosyltransferase activity	MF	0.007882608
GO:0030594	2	24	neurotransmitter receptor activity	MF	0.008919826
GO:0045202	4	133	synapse	CC	0.009410858
GO:0003676	5	192	nucleic acid binding	MF	0.012521123
GO:0000166	2	22	nucleotide binding	MF	0.012774127
GO:0098839	2	34	postsynaptic density membrane	CC	0.018704073
GO:0005344	2	12	oxygen carrier activity	MF	0.019379057
GO:0005833	2	12	hemoglobin complex	CC	0.019379057
GO:0031720	2	12	haptoglobin binding	MF	0.019379057
GO:0031838	2	12	haptoglobin-hemoglobin complex	CC	0.019379057
GO:0043177	2	12	organic acid binding	MF	0.019379057
GO:0015671	2	14	oxygen transport	BP	0.022575949
GO:0019825	2	15	oxygen binding	MF	0.023693619
GO:0072562	2	16	blood microparticle	CC	0.025104021
GO:0000287	3	97	magnesium ion binding	MF	0.029130964
GO:0043005	4	186	neuron projection	CC	0.031844946
GO:0034220	2	47	monoatomic ion transmembrane transport	BP	0.032078273

Journal of Experimental Biology • Supplementary information

GO:0048856	2	45	anatomical structure development	BP	0.033027033
GO:0004222	3	109	metalloendopeptidase activity	MF	0.033765958
GO:0006486	3	92	protein glycosylation	BP	0.037455469
GO:0006508	5	276	proteolysis	BP	0.040969444
GO:0042744	2	20	hydrogen peroxide catabolic process	BP	0.041123724
GO:0006955	4	154	immune response	BP	0.04818045

Journal of Experimental Biology • Supplementary information
